# Quantifying retention during pre-antiretroviral treatment in a large urban clinic in Uganda

**DOI:** 10.1186/s12879-015-0957-1

**Published:** 2015-07-01

**Authors:** Barbara Castelnuovo, Joseph Musaazi, Rachel Musomba, Rosalind Parkes Ratanshi, Agnes N. Kiragga

**Affiliations:** Infectious Diseases Institute, Makerere University, Kampala, Uganda

**Keywords:** Retention, Cascade, pre-ART, HIV

## Abstract

**Background:**

Retention studies are usually focused on patients on antiretroviral treatment (ART), however in Sub-Saharan Africa many patients get lost to program (LTP) in the pre-ART care period.. We investigated the proportion of patients not retained in care and factors associated with LTP (dead or lost to follow up ≥6 months) in the pre-ART care period.

**Methods:**

We analyzed data from the Infectious Diseases Institute, Kampala, Uganda. We included all adult patients ≥18 years, ART naïve at program enrollment from 1^st^/Jan/2005. We described the number of patients not retained in care during the 3 steps of enrollment-to-treatment “cascade”: Step 1) From enrollment to CD4 count testing, Step 2) ART eligibility assessment. Patients were initially considered eligible if CD4 count was <200 cell/μL, and <350 cell/μL from 2012 onwards; Step 3) From eligibility to ART start. We described cumulative probability of being LTP by gender and ART eligibility using Kaplan Meier estimates. We used a Cox proportional hazards model to identify factors associated with being LTP at any stage for all patients and for those with a CD4 count available. Factors considered were age, gender, year of enrollment, and WHO stage.

**Results and discussion:**

After enrollment in our program, cumulatively, a low proportion of patients (30.8 %) were retained and started on ART. The cumulative probability of being LTP was higher in males and patients not eligible for ART. In the multivariable Cox proportional Hazards model, male gender (HR: 1.19 CI 1.12-1.19) and clinical WHO stage 3 and 4 (HR: 1.20 CI 1.13-1.27) were associated with being LTP while older age was protective (HR: 0.98 0.96-0.99). Patients enrolled in the program more recently were also at lower risk of being LTP. In addition, among patients with CD4 count test, patients with higher CD4 count were at higher risk of being LTP.

**Conclusions:**

In our program there has been suboptimal retention of patients in pre-ART care, particularly of patients not eligible for ART. Since the proportion of eligible patients has recently increased due to the higher recommended threshold for ART eligibility (CD4 count > 500 cell/μL in 2014), this could lead to an increase in program retention as more people fall under the recommended threshold and seek care.

## Background

Lost to follow up studies are usually focused on patients on antiretroviral treatment (ART). However in Sub-Saharan Africa many patients get lost in the pre-ART care period, when they are expected to attend visits for ART screening which involve CD4 count testing and assessment for ART eligibility. The cascade from HIV testing to treatment usually consists of three steps: Step I: enrollment in care and CD4 count measurement, step II: assessment for ART eligibility, and step III: ART start [1]. Typically in Sub-Saharan African HIV care programs, it takes between 1–3 months to ensure patients are ready to initiate ART [2]. During this period, patients are counseled on ART readiness, adherence and on other informed about important aspects of ART such as toxicity and side effects.

Reviews published from resource-limited settings [1–4] and Sub-Saharan Africa in particular, show dropout rates of individuals who test HIV positive before ART initiation varying between 15 % and 60 % [1–3, 5]. In resource-limited settings where there are limited or incomplete national death registries, it is difficult to confirm whether patients who drop out are dead. In such large HIV care programs, there are limited resources to actively track patients who drop out of care especially prior to ART start. In addition, ART delivery has been rapidly decentralized however systems have not been put in place to efficiently transfer patients or to trace patients who migrate through different programs, and therefore the magnitude of patient lost to follow up could be overestimated [6].

In this study we sought to investigate the proportion of patients not retained in the program and attempted to report the reason for attrition during 9 years of offering care and treatment to HIV positive individuals, and investigated factors associated with being lost to program.

## Methods

### Study setting

The Infectious Diseases Institute (IDI), Makerere University, is an HIV center of excellence [7] located in Mulago referral Hospital in Kampala. IDI is one of the first HIV care and treatment organizations in Uganda; the IDI clinic began providing HIV care in 2002, while free antiretroviral treatment has been provided since April 2004. Similarly to other programs in the region [8], patients are assessed for ART eligibility and they are expected to attend at least two counseling session in order to be prepared to start ART.

### Study population

In this analysis we included all patients ≥18 years and ART naïve at enrollment in the IDI clinic from the 1^st^ of January 2005 to December 2013. We described the number of patients who were not retained and the reason for attrition (lost to follow-up, death or transferred to another program) during these 3 steps of enrollment-to-treatment “cascade”: 1) From enrollment to CD4 count testing, 2) ART eligibility assessment (patients who received their CD4 count at the following clinic visit). Patients were considered eligible for ART initiation according to the WHO guidelines adopted in the country: CD4 count <200 cell/μL [9] from 2005 to 2011 and <350 cell/μL in the 2012–2013 period [10]; 3) From ART eligibility to ART initiation.

### Ascertainment of outcomes

Up to 2011 ascertainment of outcomes of patients who missed clinic visits was not systematically performed; since 2011 ascertainment of patients’ outcomes has been ongoing and several attempts are often made (up to three times) to contact patients (and/or their next of kin) by phone call. However often phone contacts are not available or up to date. Patients were defined as lost to follow up (LTFU) if they do not return to the clinic for at least 6 months and they were known to be alive and disengaged from care, or if the outcome unascertainable. From June 2011, upon patients’ request or following the providers’ advice, patients were transferred out to other HIV care facilities through a structured process. This process involved counseling and provision of a referral form to be delivered to the future HIV care providers containing all the relevant health information. It is however possible that some patients transfer themselves without informing the providers.

### Statistical analysis

In the survival analysis, time at risk was calculated as the difference between date of enrolment and the following dates; Step 1: date of death (if died), or randomly assigned date within two weeks from enrolment or next scheduled appointment date when CD4 testing should have occurred; Step 2: date of CD4 blood draw; Step 3: date of last visit prior to ART initiation for patients eligible and started ART, while the date of last pre-ART visit or database closure (25^th^ July 2014) for the patients who were eligible but had not yet started ART, whichever came first. For the patients not eligible for ART, again the date of last pre-ART visit or database closure whichever came first for the patients who were retained in care, and date of ART eligibility assessment for the patients who were not retained in care. The definition of lost to program (LTP) included patients who were either dead or lost to follow-up, but excluded those who transferred out of care in the pre-ART care period. All patients who were transferred out were censored at the date of transfer. We described time from enrollment to CD4 count testing and later time to ART start and cumulative probability of being LTP by gender and ART eligibility using Kaplan Meier estimates.

We used a Cox proportional hazards model to identify factors associated with being LTP at any of the stages in the enrollment-to-treatment cascade. Factors considered were age, gender, year of enrollment in IDI (year 2008 was identified as the beginning of the rapid decentralization of HIV services in Uganda), WHO clinical stage. We performed a subgroup analysis that included only patients with a CD4 count measurement, and sought to identify time to CD4 cell count testing, as well as predictors of LTP. Missing data was observed on WHO clinical stage and multiple imputation using chained equations, with five rounds and was used to fill in the missing data. All analysis was performed using STATA corp 12.2, Texas, USA.

The protocol for the use of retrospective use of data routinely collected in IDI was reviewed and approved by the Makerere University Faculty of Medicine Research and Ethics Committee (approval number: 120–2009) and the Uganda National Council for Science and Technology (approval number: HS 683). According to the protocol procedures patients do not provide verbal or written consent, but all their information is analyzed after stripping it of unique personal identifiers.

## Results and Discussion

**Fig. 1:**
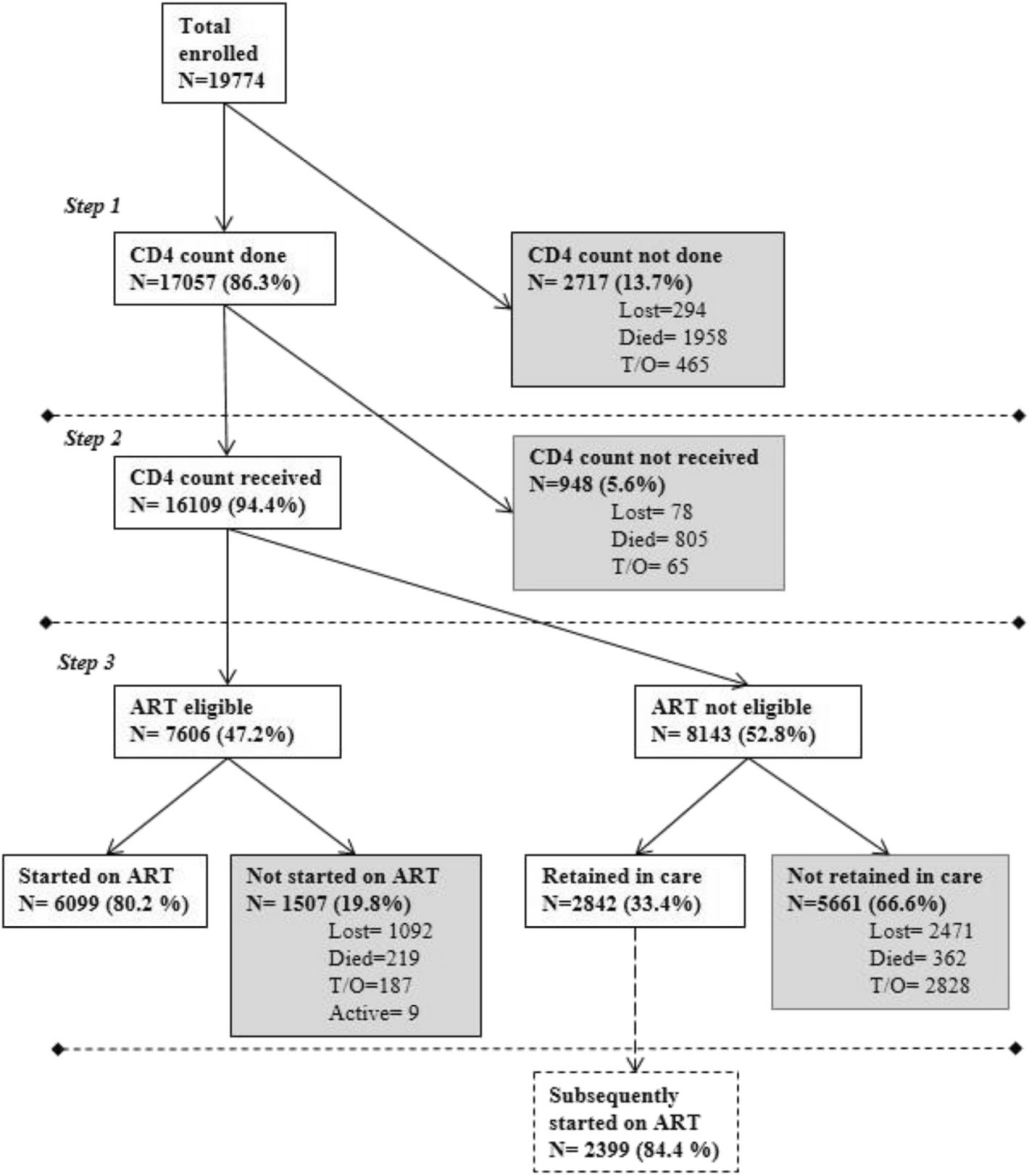
Patients retained and lost to program in the cascade from program enrollment to ART initiation

Of a total of 19,774 patients enrolled in the IDI program between January 2005 to December 2013, 12,926 (65 %) were female, the median age was 33 years (interquartile range (IQR): 27–39), and 42 % were in WHO clinical stage III and IV. Fig. 1 shows in detail the number and proportion of patients retained in care at IDI and the reason for attrition during the 3 steps of the enrollment-to- treatment cascade.

Overall of the 19,774 patients, 2,717 (13.7 %) never had a CD4 count test (Step1), of which 10.8 % were LTP; of the 17,057 retained who achieved a CD4 count test, 948 (5.8 %) did not return and therefore were not assessed for ART eligibility (Step 2). Of the 16,109 assessed for ART 7,606 (46 %) were eligible for initiation, and of these 6,099 (80.2 %) were started on ART, while the remaining patients were LTP before ART start. Overall after enrollment in our program a low proportion of patients (30.8 %) were retained and started on ART. The median time from enrollment to CD4 count testing was 25 days (IQR: 0–35) and from eligibility assessment to ART initiation was 56 days (IQR: 36–112).

**Fig. 2:**
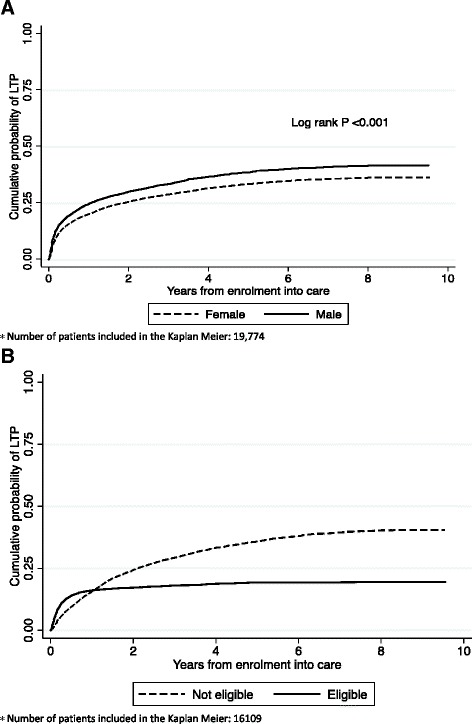
Cumulative probability of being LTP **a** by gender **b** by CD4 count ^*^

The overall cumulative probability of being LTP was 21 % (95 % CI: 20 % - 22 %) after 1 year, 30 % (95 % CI: 29 % - 31 %) after 3 years, and 35 % (95 % CI: 34 % - 36 %) after 5 years from enrolment into care. The cumulative probabilities of being LTP after 5 years was higher among males compared to females 38 % (95 % CI: 37 - 40 %), versus 33 % (95 % CI: 32 % - 34 %), *P* < 0.001 Fig. 2a, and was higher among patients not eligible for ART compared to patients eligible 36 % (95 % CI: 34 % - 37 %) versus 19 % (95 % CI: 18 %-20 %), *P* < 0.001 Fig. 2b.

In the multivariable Cox proportional Hazards model, male gender (HR: 1.19, 95 % CI: 1.12-1.19) and clinical WHO stage 3 and 4 (HR: 1.20, 95 % CI: 1.13-1.27) were associated with being LTP, while older age was protective (HR: 0.98, 95 % CI: 0.96-0.99 per 5 years increase). Patients recently enrolled in the IDI program had a lower risk of being LTP (Table 1).

**Table 1:** Factors associated with lost to program after enrolment into HIV care

	All patients ^1^			Patients with CD4 count test performed ^2^ (N = 17057)	
	(N = 19774)				
Variable	Crude HR (95 % CI)		*P*-value	AHR 95 % (CI)	*P*-value
Females	1.00			1.00	
Males	1.19 (1.12 – 1.26)		<0.001	1.23 (1.15– 1.31)	<0.001
Age, 5 year increase	0.98 (0.96 – 0.99)		0.006	0.99 (0.97– 1.00)	0.183
WHO Stage^a^ I-II	1.00			1.00	
III-IV	1.20 (1.13 – 1.27)		<0.001	1.29 (1.21 – 1.38)	<0.001
Year of enrolment					
2005 - 2008	1.00			1.00	
2009 - 2011	0.70 (0.64 – 0.75)		<0.001	1.71 (0.65 – 0.77)	<0.001
2012 - 2013	0.39 (0.31 – 0.52)		<0.001	0.19 (0.12 – 0.29)	<0.001
CD4 count 50 cell/μL increase		N/A		1.03 (1.02 – 1.03)	<0.001

1 -Predictor of LTP at any stage from enrolment into care for all patients

2 -Predictor of LTP at any stage from enrolment into care among patients with CD4 count test performed

a. 2120 patients with missing data on WHO clinical stage

N/A: not applicable

Among patients with CD4 count test assessment, factors associated with being LTP were similar (except for older age which was no longer protective): in addition patients with higher CD4 count were more likely to be LTP with an HR of 1.03 (95 % CI 1.02 – 1.03) per 50 cell/μL increase.

## Discussion

As found in other studies from Sub-Saharan Africa [1] male patients and patients who are less symptomatic or do not immediately receive ART are more likely to get LTP, probably due to their perceived low risk of AIDS. A substantial proportion of patients not retained in care, especially in the most recent years, is explained by the rapid decentralization of ART services in Uganda started in 2008, when ART was made available in all districts and at lower level facilities and many patients were transferred to clinics closer to their residence or place of work; therefore the exit from the cascade within our program may not necessarily reflect an equivalent proportions of patients from the overall cascade from HIV diagnosis to ART start [11].

The overall mortality particularly in patients eligible for ART (6.7 %) is lower, likely due to underestimation, than previously reported rates from routine programs in Uganda (28 % - 37 %) where patients were actively tracked and visited at home following disengagement from care [5, 12]. Furthermore, the availability of point of care CD4 count testing services could have led to reduction in the proportion of patients LTP at Step 1 of the cascade [13]. In our program however, patients were assessed and initiated on ART in a relatively short time period (ART eligibility within a month and ART initiation within 2 months from enrollment), and the greatest risk LTP was observed among patients who were not eligible for ART and therefore after receiving a CD4 count test (Step 2).

## Conclusions

In our program, routine tracing of patients who missed their clinic appointments was implemented in 2011. This could have made an impact in reducing pre-ART lost to care. Where feasible patients lost to program should be actively tracked and health information systems used to monitor patients’ migration between facilities so to avoid overestimation of the number of patients LTP. Since healthier patients are at higher risk of getting LTP, it is imperative that emphasis is given in training health workers to efficiently deliver motivation messages and to enhance health-seeking behavior. In our program patients eligible for ART are referred for ART preparation counseling, while those not eligible are simply given a follow up clinic appointment. Programs should consider referring as well patients not eligible for ART to post CD4 count assessment counseling in order to emphasize the importance of reaming engaged in care.

The reduction in LTP over time could also be attributed to the change in WHO guidelines [14] and the recommendation to start ART at higher CD4 counts (from 200 to 350 cell/μL) since patients who don’t receive immediately ART are at risk of getting LTP; in our program we recently (August 2014) lifted the threshold to 500 cell/μL and this could potentially lead to an increase in program retention rates.

One potential limitation of our results could be due to the likelihood of differential lost to follow-up long the years, which can lead to difficulties in the classifications of patients along the cascade and a potential for bias in the estimates of risk factors. Overall, we feel that our results are generable to similar settings.
